# Effects of Traditional Chinese Medicine for Vaginal Lavage Combined with Psychological Intervention in Postoperative Patients with Cervical Cancer

**DOI:** 10.1155/2021/5751795

**Published:** 2021-12-15

**Authors:** Rufen Ma, Ruixiang Yu, Yuchao Yuan, Baofeng Ren, Yuan Li

**Affiliations:** ^1^Department of Women Health Care, Zhangqiu Maternity and Child Care Hospital, Jinan 250200, China; ^2^Department of Women Health Care, Yantaishan Hospital, Yantai 264000, China; ^3^Department of Obstetrics and Gynecology, Zhangqiu District People's Hospital, Jinan 250200, China; ^4^Medical Insurance Department, Zhangqiu District People's Hospital, Jinan 250200, China; ^5^Health Care Department, Zhangqiu Maternity and Child Care Hospital, Jinan 250200, China

## Abstract

**Purpose:**

To explore the effects of traditional Chinese medicine for vaginal lavage combined with psychological intervention on the immune function and clinical efficacy in patients with cervical cancer.

**Methods:**

Patients with cervical cancer treated in our hospital from January 2020 to May 2021 were included in this study. All patients were treated with traditional Chinese medicine for vaginal lavage combined with psychological nursing intervention. The treatment outcomes of the patients were observed, and the quality-of-life scores and depression of the patients before and after treatment were compared. Changes in T-lymphocyte subset-related indicators, changes in blood routine-related indicators, and changes in the detection level of tumor markers were compared with anxiety scores.

**Results:**

After treatment, depression and anxiety were significantly reduced and the patient's quality of life significantly improved. After treatment, the patient's CD3^+^, CD4^+^, and CD4^+^/CD8^+^ proportions were dramatically higher than before treatment (*P* < 0.05), there was no significant difference in CD8^+^ proportion before and after treatment (*P* > 0.05), and the white blood cell (WBC), hemoglobin (Hb), platelet (PLT) of patients, and the level of tumor marker (CA125) after treatment were immensely lower than before treatment (*P* < 0.05).

**Conclusions:**

Treating patients with cervical cancer with traditional Chinese medicine for vaginal lavage combined with psychological nursing can effectively improve the patient's immune function, effectively reduce the level of tumor marker CA125, increase the level of T-lymphocyte subsets, and improve the bone marrow hematopoietic function.

## 1. Introduction

Cancer is a leading condition that threatens human health and life, and its incidence and mortality are rapidly increasing. There are 4.29 million new cancer cases every year, accounting for 20% of the world's share, and 2.81 million deaths. The prevention and treatment of cancer are urgent needs [1]. Gynecological malignant tumors also have the characteristics of high morbidity and high mortality in the world, and their tendency in youth has become more evident in recent years, which seriously threatens the life and health of women. The incidence of cervical cancer ranks second among gynecological malignancies in the world [2, 3]. According to the World Health Organization data, there are 530,000 new cases each year, and about 250,000 women die from cervical cancer. In China, there are about 140,000 new cases of cervical cancer and about 37,000 deaths each year, which is a high incidence area for cancer [4–7].

Currently, chemotherapy is commonly used in cervical cancer treatment. Chemotherapy before surgery can not only reduce the choice of surgery but also increase the resection rate of surgery, thereby controlling tumor development and prolonging the survival of patients. However, chemotherapy has a relatively large number of adverse reactions in patients. It kills cancer cells in the patient's body and also has a more significant impact on normal cells. It is evident that it reduces the immune function of the patient's body [8]; the patient has symptoms such as decreased white blood cells, anemia, and loss of appetite [9]. In recent years, Chinese medicine has been widely used in the treatment of malignant tumors. After analyzing the pathogenesis of cervical cancer, oral Chinese medicine decoction for patients with cervical cancer can not only effectively improve the bone marrow suppression but also enhance the immune function of the patients. The adverse reactions caused by Chinese medicine are relatively lesser.

Previous medical research shows that the body's antitumor immune response mechanism is complex and delicate. The occurrence and development of tumors are closely related to the body's immune state. The body's immune system can eliminate tumor cells in the body that are sensitive to immune responses. Tumor cells escape the recognition, surveillance, and attack of the body's immune system and then continue to divide and proliferate through various mechanisms, which is also the main reason for the formation, development, and metastasis of tumors in the body. Cellular immunity mediated by T lymphocytes is the main aspect of tumor immunity. Abnormal numbers and functions can allow tumor cells to escape host immune surveillance [10]. Therefore, the detection of T-cell subsets has extremely high application value in the evaluation of clinical efficacy and prognosis of tumors, and its functional status directly reflects the body's ability to resist the invasion of cancer cells [11, 12]. Generally, the constant ratio of CD4^+^/CD8^+^ maintains the balance of cellular immunity. If the balance between the CD4^+^ and CD8^+^ is broken, the immunity will be suppressed and the mutated tumor cells will not be recognized [13].

Due to factors such as lack of female characteristics caused by diseases and treatments, it is difficult for more gynecological malignant tumor patients to accept the status quo in a short time and thus psychological problems mainly manifested by depression and anxiety symptoms. Faced with the threat of disease, patients with malignant tumors are under physical, emotional, and social pressures during diagnosis and treatment, resulting in severe mental health problems. Some patients in a state of depression are associated with malignant tumors, and physical pain and mental illness increase the pain of patients. Pieces of evidence show that the incidence of depression in patients with gynecological malignancies is 12%–23% [14–16]. The presence of depression and anxiety will complicate the treatment of tumors and reduce the patient's compliance with treatment, thereby affecting the outcomes of therapy. With the transformation of the “biopsychosocial medical model,” the important influence of psychological factors such as depression and anxiety on the treatment of malignant tumors has attracted more and more attention from experts and scholars.

Psychological intervention treatment is standardized nursing methods including evidence-based medicine, holistic nursing, health education, and continuous quality improvement. Timeliness and sequentiality are its distinguishing characteristics. The premise of path implementation is first to set up a clinical care pathway team and then to formulate a corresponding care pathway plan, so that patients can obtain the best-quality service, reduce the occurrence of complications, promote patient recovery and shorten the length of hospitalization, and reduce the waste of medical resources [17, 18].

The World Health Organization launched a global initiative: to expand prevention, screening, and treatment interventions and treat the elimination of cervical cancer as a public health issue in the 21st century. There are no Western medicines and vaccines with proven efficacy for the current postoperative treatment of cervical cancer, usually given local application of interferon treatment. In order to improve the symptoms of the disease, hinder the progress of the disease, and improve the quality of life of the patients, our hospital now adopts traditional Chinese medicine for vaginal lavage combined with psychological intervention to treat patients with high-risk HPV infection of the cervix.

## 2. Materials and Methods

### 2.1. Research Objects

All cases were diagnosed and treated at the Department of Oncology and Gynecology of Zhangqiu Maternity and Child Care Hospital, Jinan, Shandong, China. The patients (96 cases) who were diagnosed for the first time and underwent postoperative treatment of cervical cancer from January 1, 2020, to May 31, 2021, were included in the study group. There was complete clinical data, including 51 married cases, 45 unmarried cases, age range from 23 to 58 years old, with an average age of (38.11 ± 12.33) years, 86 cases of nonmenopause, and 10 cases of menopause. This study was approved by the ethics committee of the Zhangqiu Maternity and Child Care Hospital, and all patients provided written informed consent.

#### 2.1.1. Inclusion Criteria

1. Patients with a history of sexual life
2. There was no history of cervical conization and hysterectomy
3. There was no vaginal medication within 2 weeks before treatment and no intercourse within 3 days
4. Individuals diagnosed with cervical cancer through various clinical examinations and have undergone surgical treatment
5. Have no history of mental illness or mental illness that affects informed consent, have certain communication and language skills, and exclude critically ill patients with severe illnesses
6. No other malignant tumors and severe comorbidities
7. Those who have surveyed questionnaires and have complete medical information

#### 2.1.2. Exclusion Criteria

1. Age <20 years old or > 65 years old
2. Those with asexual life history
3. Those who do not meet the diagnostic criteria
4. Patients with cervical intraepithelial lesions and cervical cancer
5. Patients with severe heart, liver, kidney, blood, or endocrine system and other primary diseases, mental diseases, and immune diseases
6. Breastfeeding women and pregnant women
7. Incomplete medical information

### 2.2. Research Methods

#### 2.2.1. Treatment Methods

Every night, 200 mL decoction of traditional Chinese medicine (20 g of *Sophora flavescens*, 20 g of *Cnidium monnieri*, 20 g of smilacis glabrae rhizoma, 20 g of densefruit pittany root bark, 20 g of kochiae fructus, 20 g of herba hedyotidis, and 20 g of scutellariae barbatae herba) was administered for vaginal lavage, followed by vaginal application of recombinant human interferon ɑ-2b vaginal effervescent tablets (Beijing Kaiin Technology Co., Ltd., S20120019). All patients were treated once a day for 10 consecutive days as a course of treatment. The next course of treatment was performed after 3 days of clean menstruation in the next month. The menopausal patients were treated for 10 consecutive days each month for a total of 3 courses of treatment. During treatment, if the patient has sex, condoms must be used. There were no noticeable adverse reactions in patients during the medication.

*(1) Psychological Intervention*. In the treatment visits, targeted psychological counseling and professional psychological interventions were provided to patients, and patients were patiently listened regarding their main complaints. Nurses use standardized reception language for patients, check the relevant information of the patients, ask the patients' inner feelings, and help the patients as much as possible to relieve the inner tension and anxiety.

The self-rating anxiety scale (SAS) and the self-rating depression scale (SDS) were applied to correctly assess the patients' mental state and guide them to talk. At the same time, the nurses patiently answer to alleviate patients' anxiety, fear, low self-esteem, and other psychologies. Effective communication was conducted with the patient's family members to understand and care more about the patient, reduce the patient's negative emotions, and increase confidence in recovery. After the operation, the patients were asked about their feelings, they were regularly asked about their feelings, and music sedation and analgesia intervention were given to reduce the patient's psychological stress after surgery.

#### 2.2.2. Detection Indicators

*(1) Zung Self-Rating Depression Scale (SDS)*. The scale adopts a 4-level scoring system to assess the frequency of symptoms. The criteria are as follows: “1” no or very little time, “2” a little time, “3” a lot of time, and “4” most of the time or all of the time. Result analysis: the scores for each of the 20 items are added together to obtain the total crude score. The normal upper limit of the reference value for the total raw scores is 41, and the standard score is the integer portion of the total raw scores multiplied by 1.25. The higher the score, the more severe the symptoms in this area. The standard score is as follows: mild depression: 53–62, moderate depression: 63–72, and major depression: >72. The cutoff value is 53 points.

*(2) Zung Self-Rating Anxiety Scale (SAS)*. The main statistical index of SAS is the total score. The score of each of the 20 items is added up to get the crude score. The higher the standard score is, the more serious the symptoms are. According to the Chinese norm SAS standard score, the total anxiety score below 50 is normal. Those with 50–59 scores were mild, those with 60–69 scores were moderate, and those with 70 or above scores were severe.

*(3) Changes of T-Lymphocyte Subset-Related Indicators (CD3^+^, CD4^+^, CD8^+^, and CD4^+^/CD8^+^) and blood routine-related indicators*. Before and after treatment, 5 mL of the patient's fasting venous blood was taken and placed on the automatic blood separator of our hospital for centrifugation, at 3,000 rpm, centrifuged for 10 min. The supernatant was taken and stored at −20°C for examination. Before and after treatment, the counts of white blood cell (WBC), hemoglobin (Hb), platelet (PLT) in patients were detected.

*(4) Coagulation Function*. Before and 5 days after treatment, 6 mL elbow venous blood of patients in the morning was taken and centrifuged at 3,500 rpm for 10 min. Then, the supernatant was taken, and the coagulation function and the changes in fibrinogen (FIB) and D-dimer (D-D) were detected by a fully automatic biochemical analyzer (American Beckman AU5800).

The normal reference value of plasma FIB was 1.8–3.5 g/L, and the measured value (>3.5 g/L) was an outlier. The normal reference value of plasma D-D was 0–0.55 *μ*g/mL, and the measured value (>0.55 *μ*g/mL) was an outlier, which was clinically proven effective when the FIB D-D value decreased.

*(5) Tumor Marker (CA125) Examination*. A total of 3 mL venous blood was collected on an empty stomach in all patients within 2 weeks before the operation, the serum was separated after centrifugation, and the level of serum CA125 was measured by the chemiluminescence method with Roche E70 automatic immunoanalyzer.

### 2.3. Statistical Methods

The detection data of all patients were expressed as mean ± SD using SPSS 22.0 statistical analysis software. One-way ANOVA test or chi-square test was used to compare the differences between different groups. *T*-test was used to test the data between the same groups. *P* < 0.05 was considered statistically significant.

## 3. Result

### 3.1. Clinical Characteristics of Patients

In this study, 96 cases of postoperative cervical cancer were selected, including 51 married patients and 45 unmarried patients, aged from 23 to 59 years old, with an average age of (38.11 ± 12.33) years old, 86 premenopausal patients, and 10 postmenopausal patients. According to the International Federation of Gynecology and Obstetrics (FIGO) classification in 2009, there were 43 patients with stage I-II cervical cancer, aged between 23 and 56 years, with an average age of 37.38 ± 6.82 years, and 53 patients with stage III-IV cervical cancer, aged between 25 and 59 years, with an average age of 40.5 ± 7.15 years, as shown in Table 1.

### 3.2. Postoperative Depression and Anxiety in Patients with Cervical Cancer

**Figure 1:**
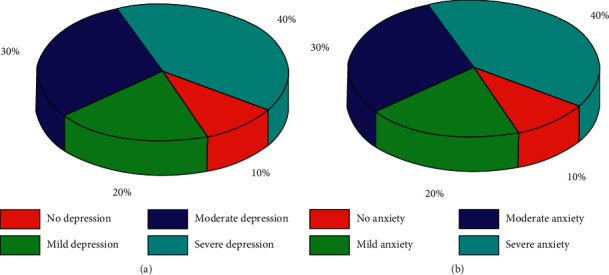
Postoperative depression and anxiety in patients with cervical cancer. (a) The score of SDS. (b) The score of SAS.

The mean standard score of SDS in 96 patients with cervical cancer after the operation was 47.2 ± 10.3. According to SDS standard score, there were 17 patients with depression, accounting for 17.7%, and 79 patients without depression, accounting for 82.3%. According to the standard of depression degree, 11 people were mildly depressed, accounting for 11.4%, 4 people were moderately depressed, accounting for 4.2%, and 2 people were severely depressed, accounting for 2.1%, as shown in Figure 1(a). The mean standard score of SAS in 96 patients with cervical cancer after the operation was 41.7 ± 9.2. According to the SAS standard score, there were 21 patients (21.9%) with anxiety, 75 patients (78.1%) without anxiety, 15 patients (15.6%) with mild anxiety, 5 patients (5.2%) with moderate anxiety, and 1 patient (1.1%) with severe anxiety, as shown in Figure 1(b).

### 3.3. The Comparison of SAS, SDS, and KPS

**Figure 2:**
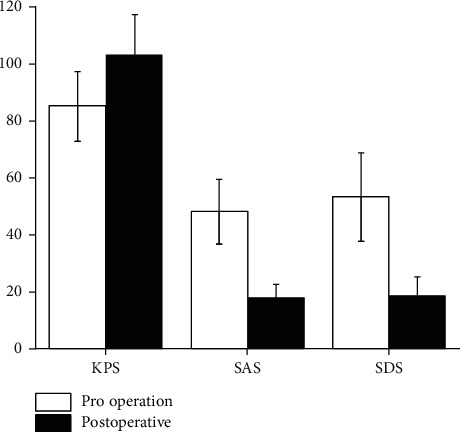
Comparison of psychological and quality-of-life scores before and after treatment.

Before and after treatment, the scores of SAS, SDS, and KPS were compared, as shown in Figure 2. Before psychological care, the scores of SAS, SDS, and KPS were all poor in patients with cervical cancer, and the intragroup difference was not statistically significant (*P* > 0.05). After psychological nursing, the patients' SAS, SDS, and KPS scores were improved, and there was a significant difference in patients with cervical cancer before and after the treatment (*P* < 0.05), indicating that Chinese medicine for vaginal lavage combined with psychological intervention can effectively improve the quality of life of patients with cervical cancer. Psychological intervention refers to basic nursing for patients with cervical cancer, while paying more attention to the condition of the patient, which is conducive to the rehabilitation of the patient, can effectively improve the patient's unhealthy mentality and improve the quality of life of the patient, which is a high-quality nursing guarantee.

### 3.4. Comparison of T-Lymphocyte Subsets

Although postoperative radiotherapy and chemotherapy for cervical cancer can prolong disease-free survival and reduce mortality, most patients have noticeable adverse reactions and suppressed immune function. Immunological studies have shown that the occurrence, development, and prognosis of tumors directly impact the immune function of the body, especially T-cell-mediated cellular immunity. T cells mainly include CD4^+^ and CD8^+^ subgroups. Traditionally, CD4^+^ T cell is considered as the helper T cell, and CD8^+^ T cell is regarded as a suppressor T cell. Therefore, the proportion of CD4^+^, CD8^+^, and CD4^+^/CD8^+^ cells can directly reflect the cellular immune function of patients with cervical cancer. The proportion of CD4^+^ was 38.82 ± 4.55 before treatment and 52.98 ± 4.28 after treatment, and the proportion of CD8^+^ and CD4^+^/CD8^+^ was 34.01 ± 6.42 and 1.14 ± 0.15 before treatment and 32.24 ± 5.73 and 1.56 ± 0.16 after treatment, respectively, as shown in Table 2. The results showed that the level of CD3^+^, CD4^+^, and CD4^+^/CD8^+^ significantly increased. In contrast, the level of CD8^+^ dramatically decreased after treatment compared with that before treatment (*P* < 0.05), suggesting that traditional Chinese medicine for vaginal lavage can effectively enhance the immune function of postoperative patients with cervical cancer.

### 3.5. The Comparison of Blood Routine Indexes of 96 Patients before and after Treatment

The counts of WBC, Hb, and PLT in patients with cervical cancer before postoperative treatment were 7.18 ± 1.10 10^9^/L, 120.55 ± 10.02, and 214.37 ± 10.14 10^9^/L. After vaginal lavage combined with psychological intervention, WBC, Hb, and PLT counts were 5.34 ± 1.05 10^9^/L, 100.18 ± 7.70 g/L, and 159.90 ± 9.39 10^9^/L, respectively, as shown in Table 3. The levels of WBC, Hb, and PLT were enormously lower after treatment than that before treatment (*P* < 0.05), suggesting that the vaginal lavage of traditional Chinese medicine can effectively improve the hematopoietic function of bone marrow in patients with cervical cancer.

### 3.6. The Changes of FIB and D-D before and after Chinese Medicine Treatment

**Figure 3:**
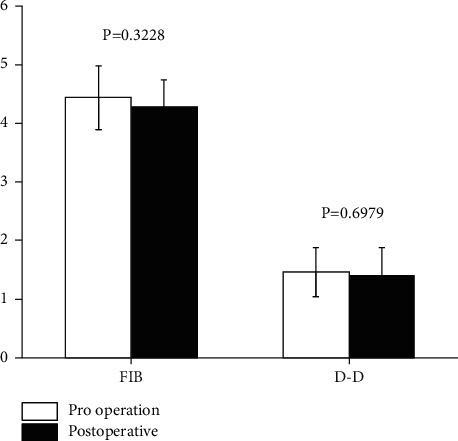
The comparison of FIB and D-D before and after Chinese medicine treatment.

It has been reported that abnormal coagulation mechanisms and fibrinolytic system caused by malignant tumors are the most important reasons leading to blood hypercoagulability and coagulation dysfunction. The coagulation function of patients with cervical cancer is increased, which is manifested in the increase of D-D and FIB. For the high coagulation value of FIB and D-D after the operation of cervical cancer, vaginal perfusion of traditional Chinese medicine can play an effective role in reducing FIB and D-D (*P* < 0.05), as shown in Figure 3.

### 3.7. The Comparison of Serum Tumor Marker (CA153) before and after Treatment

Tumor markers are substances synthesized and released by tumor cells or released by the host to reflect the existence and growth of tumors in the process of tumor genesis and development. They are related to the severity of the disease and tumor size or stage, can monitor the therapeutic effect of tumor, and can monitor the recurrence and prognosis of tumor. CA125 is the best indicator for detecting cervical cancer recurrence, and it has certain clinical significance for the follow-up of cervical cancer.

**Figure 4:**
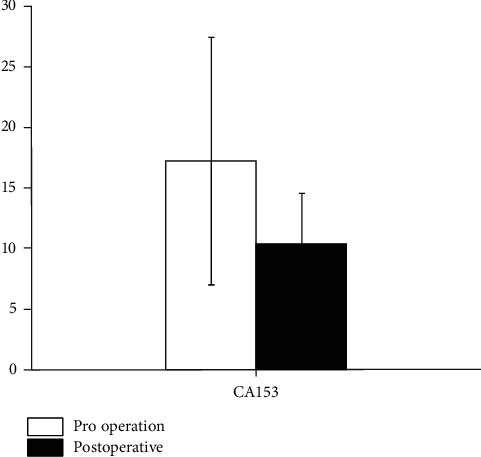
The comparison of serum tumor marker (CA153) before and after treatment.

The comparison of serum tumor marker (CA153) before and after treatment is shown in Figure 4. By t-test, the level of CA153 in patients with cervical cancer before treatment decreased from 17.23 ± 10.23 to 10.34 ± 4.23. There was an apparent difference in CA153 level before and after treatment (*P* > 0.05), suggesting that traditional Chinese medicine for vaginal lavage combined with psychological intervention can reduce serum tumor indexes.

## 4. Discussion

Cervical cancer is the fourth most common cancer in women. It is a serious condition that threatens women's life and physical and mental health. At present, surgical treatment is one of the effective treatments for cervical cancer. However, comprehensive treatment principles such as radiation therapy, chemotherapy, endocrine therapy, biological therapy, and traditional Chinese medicine should be considered after surgery. Surgery or radiotherapy and chemotherapy are a heavy blow to the human body, and many complications often occur. In traditional Chinese medicine theory, surgical treatment consumes the healthy qi and damages the body fluid [19–21], so the therapeutic principle is to strengthen the body resistance to eliminate pathogenic factors.

This study is based on the application of interferon plus vaginal lavage with traditional Chinese medicine treatment. Chinese medicine has the function of clearing heat toxins, eliminating dampness and arresting leukorrhea, and killing ascarid [22, 23]. Pharmacological studies show that matrine in Sophora flavescens can effectively sterilize without damaging normal vaginal flora [24]. The dione, dictamnine, and obacunone in Cortex Dictamni have more potent antibacterial, anti-inflammatory, antianaphylaxis, anticancer, and insecticidal pharmacological activities [25–27]. Cnidium cnidii, fructus kochiae, and rhizome smilacis glabrae have anti-inflammatory, antiviral, and antitumor effects [28–30], and the therapeutic effects are mainly achieved by promoting the apoptosis of corresponding cells. Spreading hedyotis herb contains flavonoids, iridoids, terpenoids, and anthraquinones, which can inhibit the growth of tumor cells and induce their apoptosis [31]. Scutellaria barbata D. Don contains diterpenes and its lactone, flavonoid, polysaccharides, etc., which have the pharmacological effects of promoting cellular immune function, anticancer, antivirus, antioxidation, and antiaging [32].

The results of this study showed that the percentage of CD4^+^ and CD3^+^ cells and the ratio of CD4^+^/CD8^+^ in patients treated with vaginal Chinese medicine infusion were significantly increased compared with those before treatment (*P* < 0.05). Better therapeutic outcomes have been achieved, and no adverse reactions were observed during the whole treatment process. This study adopts that the vaginal Chinese medicine perfusion can significantly improve the cellular immunity function of the organism, effectively hamper the levels of tumor marker CA125, reduce the side effects of chemotherapy, improve the bone marrow hematopoietic function, and can prevent cervical cancer recurrence and metastasis. However, there is still a gap to improve this potential concept. The insufficient sample size may lead to a large probability of error in data deviation, so we hope to increase the sample size in future research to reduce the deviation of results. In addition, the reasons for the decrease in adverse drug reactions shall be further explored to establish the efficacy of drugs better. These are the directions of our follow-up and improvement to find a better treatment for this disease.

Psychological intervention is the basic nursing of patients to pay more attention to the patient's state, conducive to the rehabilitation of patients with cervical cancer after surgical treatment. It could improve the patient's unhealthy mentality. Vaginal traditional Chinese medicine perfusion can directly act on the affected part of the body, and the clinical application is simple. Combined with psychological intervention, it is more effective to improve patients' quality of life, strengthen the treatment efficacy, and improve patient's compliance.

## 5. Conclusions

In conclusion, the patient's quality of life was significantly improved, depression and anxiety were reduced, the patient's CD3+, CD4+, and CD4+/CD8+ indicators after treatment were higher than before treatment, and the patient's WBC, Hb, and PLT counts after treatment were lower than before treatment. The treatment of patients with cervical cancer with traditional Chinese medicine for vaginal lavage combined with a psychological nursing intervention program can effectively improve the patient's immune function, lower the level of tumor marker CA125, increase the level of T-lymphocyte subsets, and improve bone marrow hematopoietic function.

## Figures and Tables

**Table 1:** Clinical characteristics of patients.

Clinical characteristics		Proportion of patients
Age	<35	12 (12.5%)
	35–60	84 (87.5%)

Marital status	Married	51 (53.1%)
	Unmarried	45 (46.9%)

FIGO stage	I-II	43 (44.8%)
	III-IV	53 (55.2%)

**Table 2:** The comparison of T-lymphocyte subsets before and after treatment.

Time	CD3^+^	CD4^+^	CD8^+^	CD4^+^/CD8^+^
Before treatment	54.85 ± 5.62	38.82 ± 4.55	34.01 ± 6.42	1.14 ± 0.15
After treatment	63.71 ± 6.54	52.98 ± 4.28	32.24 ± 5.73	1.56 ± 0.16
*P*	<0.001	<0.01	>0.05	>0.05

**Table 3:** The comparison of blood routine indexes of 96 patients before and after treatment.

Time	WBC (10^9^/L)	Hb (g/L)	PLT (10^9^/L)
Before treatment	7.18 ± 1.10	120.55 ± 10.02	214.37 ± 10.14
After treatment	5.34 ± 1.05	100.18 ± 7.70	159.90 ± 9.39
*P*	<0.05	<0.05	<0.001

## Data Availability

The data used to support the findings of this study are available from the corresponding author upon request.
